# Breast Cancer Metastases to Atypical Locations: Clinical Insights From Four Cases

**DOI:** 10.7759/cureus.99577

**Published:** 2025-12-18

**Authors:** Noor M Fazaldad, Zakiya Al-Ajmi, Nassra Al-Harthy, Samya Al-Salhi, Suad Al-Duwaiki

**Affiliations:** 1 Diagnostic Radiology, Oman Medical Specialty Board, Muscat, OMN; 2 Laboratory Medicine and Pathology, Royal Hospital, Muscat, OMN; 3 Diagnostic Imaging and Interventional Radiology, Royal Hospital, Muscat, OMN

**Keywords:** 18f-fdg-pet/ct, breast cancer outcomes, immuno-histochemical, parotid mass, rare metastases

## Abstract

Metastatic breast cancer remains the leading cause of cancer-related mortality among women. Common metastatic sites include the lung, liver, bone, lymph nodes, and brain, with distant metastases occurring more frequently than local or regional recurrences. This case series aims to highlight and raise awareness of rare metastatic sites of breast cancer, thereby promoting early detection and guiding optimal management, which may improve patient survival.

Four patients were identified with metastases to rare anatomical sites: the parotid gland, thyroid, stomach, and colon. All cases were confirmed by immunohistochemistry, with tumor cells positive for CK7 and GATA-3, consistent with breast origin. ^18^F-FDG PET/CT was instrumental in identifying hypermetabolic lesions, though differentiation from primary tumors relied on pathological evaluation. Systemic therapy, including chemotherapy, hormonal therapy, and HER2-targeted treatment, constituted the mainstay of management, with surgical intervention reserved for palliation or local control. Breast cancer metastases to rare sites pose significant diagnostic challenges, often mimicking synchronous primary tumors of the affected organs. Even with advances in oncology care, such metastases may be overlooked unless actively considered. Radiologists and clinicians should maintain a high index of suspicion for atypical metastases in patients with a history of breast cancer. Any new or enlarging lesion in an unusual site should be considered metastatic until proven otherwise. Early detection facilitates timely intervention and may improve patient prognosis.

## Introduction

Breast cancer is the most prevalent malignancy among women worldwide, with more than one million new cases diagnosed annually, and remains the leading cause of cancer-related death in this population [1]. Common metastatic sites include the lung, liver, bone, lymph nodes, and brain, with distant metastases occurring more frequently than local or regional recurrences [1].

Despite substantial progress in diagnostic modalities, surgical techniques, perioperative care, and both local and systemic adjuvant therapies, the majority of cancer-related mortality remains attributable to metastases that are refractory to conventional treatment [2]. Metastatic progression is a sequential and selective process incorporating stochastic elements and represents the culmination of complex interactions between disseminated tumor cells and host microenvironmental factors [3].

Advances in imaging and pathological assessment have facilitated the detection of metastases at less typical anatomical locations, leading to an increasing number of reports of such presentations in the literature [3].

Because these metastatic patterns are so uncommon, the prognosis and survival rates for rare breast cancer metastases are not well established. Some studies suggest that 3-30% of patients with distant metastases may experience long-term survival when treated with multiple therapeutic modalities, while other reports have been unable to define overall survival outcomes in metastatic breast cancer [2]. Current evidence indicates that the overall extent of systemic disease is a more significant predictor of survival in patients with metastatic breast cancer [2-4].

Herein, we report four cases of breast cancer metastasizing to uncommon sites, diagnosed at a tertiary care hospital. The aim is to raise clinical awareness of these unusual metastatic patterns to facilitate timely diagnosis and appropriate management, which may ultimately improve patient outcomes.

## Case presentation

Case 1: parotid gland metastasis

**Figure 1:**
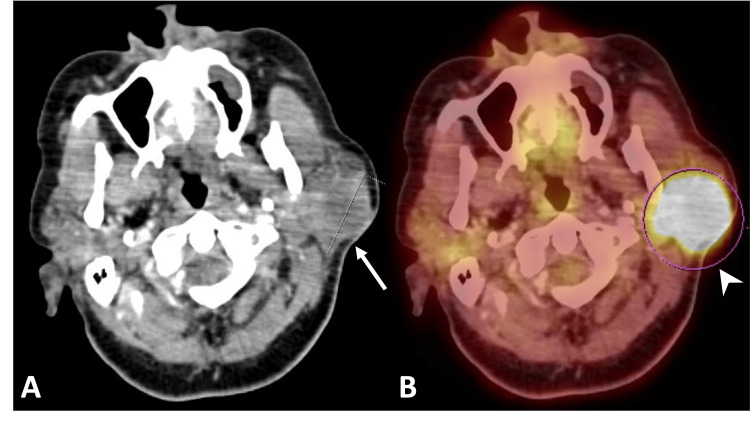
Breast cancer metastasis to the parotid gland. (A) Axial contrast-enhanced CT of the neck shows a large, lobulated, heterogeneously enhancing mass (white arrow) involving the superficial lobe of the left parotid gland. (B) 18F-FDG PET-CT demonstrates that the mass is markedly FDG-avid/hypermetabolic (arrowhead), with a maximum standardized uptake value (SUVmax) of 20. ^18^F-FDG PET-CT: Fluorine-18 fluorodeoxyglucose positron emission tomography/computed tomography; CT: Computed tomography; SUVmax: Maximum standardized uptake value

A 64-year-old woman with no significant past medical history presented with progressive left cheek swelling. Initial CT showed a large, heterogeneous, lobulated mass involving the superficial lobe of the left parotid gland (Figure 1).

**Figure 2:**
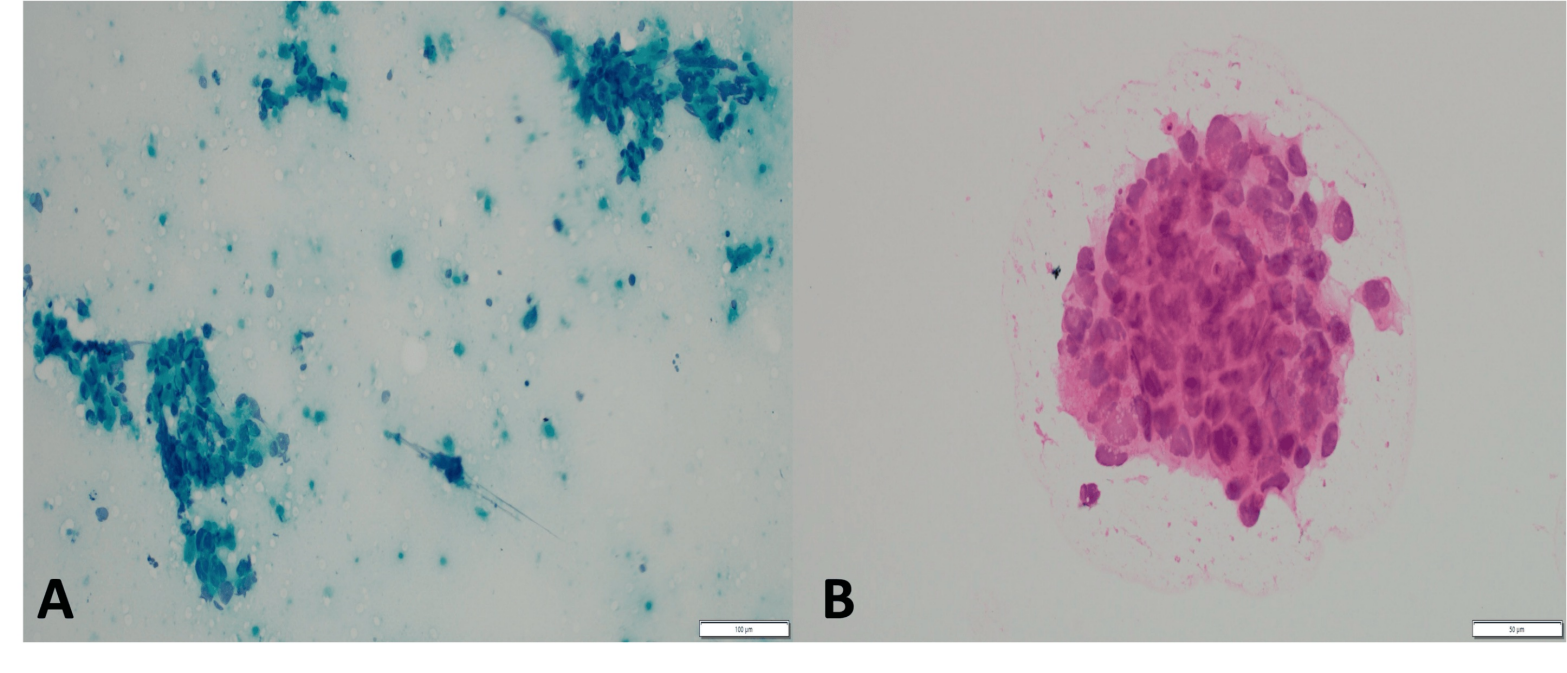
Cytomorphology of metastatic breast carcinoma in the parotid gland. (A) Papanicolaou-stained smear from the parotid gland (20× objective) demonstrating metastatic breast carcinoma with scattered aggregates and singly dispersed malignant cells. (B) Hematoxylin and eosin-stained smear from the same case (40× objective) showing markedly pleomorphic tumor cells with numerous apoptotic bodies.

**Figure 3:**
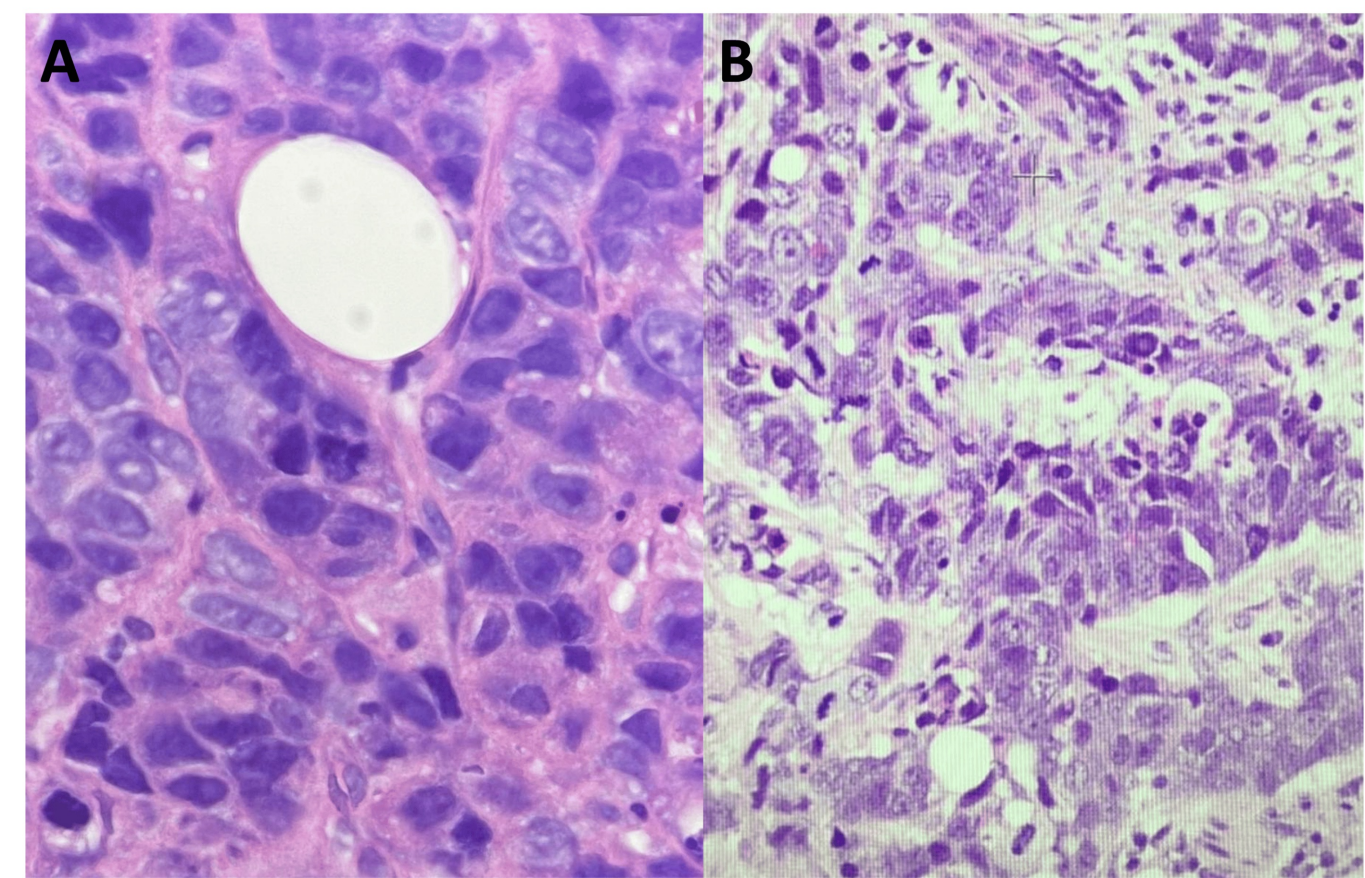
Histopathologic features of parotid metastasis from breast carcinoma. (A) Hematoxylin and eosin-stained section (40× magnification) of the primary breast carcinoma. (B) Hematoxylin and eosin-stained section of the parotid lesion showing a poorly differentiated adenocarcinoma with morphology closely resembling the primary breast tumor in Panel A, consistent with metastatic involvement of the parotid gland.

^18^F-FDG PET/CT revealed avid uptake in the parotid mass (Figure 1) as well as a hypermetabolic spiculated lesion in the right breast, associated skin thickening, and multiple enlarged right supraclavicular, axillary, and internal mammary lymph nodes. Mammography showed diffuse right breast asymmetry with pleomorphic microcalcifications, architectural distortion extending to the nipple, and nipple retraction. No prior routine screening mammography was available, making it difficult to determine the exact time of breast cancer onset. Ultrasound demonstrated a corresponding breast mass with satellite nodules and suspicious lymphadenopathy. Biopsy of the right breast lesion and axillary lymph nodes confirmed invasive carcinoma with nodal metastases. Excisional biopsy of the parotid gland mass revealed high-grade carcinoma consistent with metastasis from the breast primary (Figures 2, 3). The primary breast carcinoma was high grade, HER2 positive, ER/PR negative and demonstrated loss of GATA-3 expression. The parotid gland lesion exhibited an analogous immunophenotypic profile. Furthermore, hematoxylin and eosin-stained sections from the parotid lesion revealed morphologic features closely recapitulating those of the primary breast carcinoma. In aggregate, the morphologic and immunohistochemical findings were consistent with metastatic involvement of the parotid gland by the patient’s known primary breast carcinoma. The patient commenced chemotherapy and hormonal therapy. Follow-up ^18^F-FDG PET/CT demonstrated a significant reduction in both the parotid gland mass and breast lesion, with imaging features consistent with treatment response.

Case 2: thyroid metastasis

A 37-year-old woman with a history of right breast locally advanced invasive ductal carcinoma was previously treated with modified radical mastectomy, latissimus dorsi flap reconstruction, neoadjuvant chemotherapy, radiotherapy, and hormonal therapy. She presented with a one-month history of a palpable lump in the left breast accompanied by generalized body ache.

**Figure 4:**
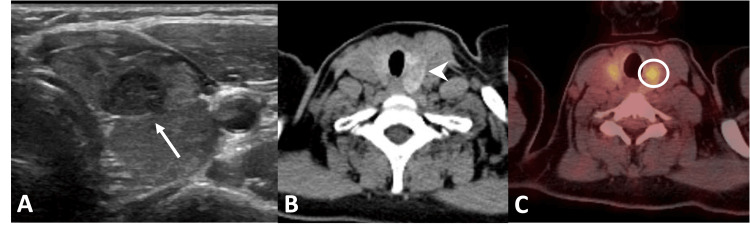
Breast cancer metastasis to the thyroid gland. (A) Gray-scale ultrasound of the left thyroid lobe demonstrates a well-circumscribed, heterogeneous, hypoechoic solid nodule (white arrow). (B) Axial unenhanced CT shows a heterogeneous hypodense nodule (arrowhead) in the left thyroid lobe. (C) Axial 18F-FDG PET-CT demonstrates mild FDG uptake within the nodule (circle), consistent with metastatic involvement. ^18^F-FDG PET-CT: Fluorine-18 fluorodeoxyglucose positron emission tomography/computed tomography; CT: Computed tomography; FDG: Fluorodeoxyglucose

**Figure 5:**
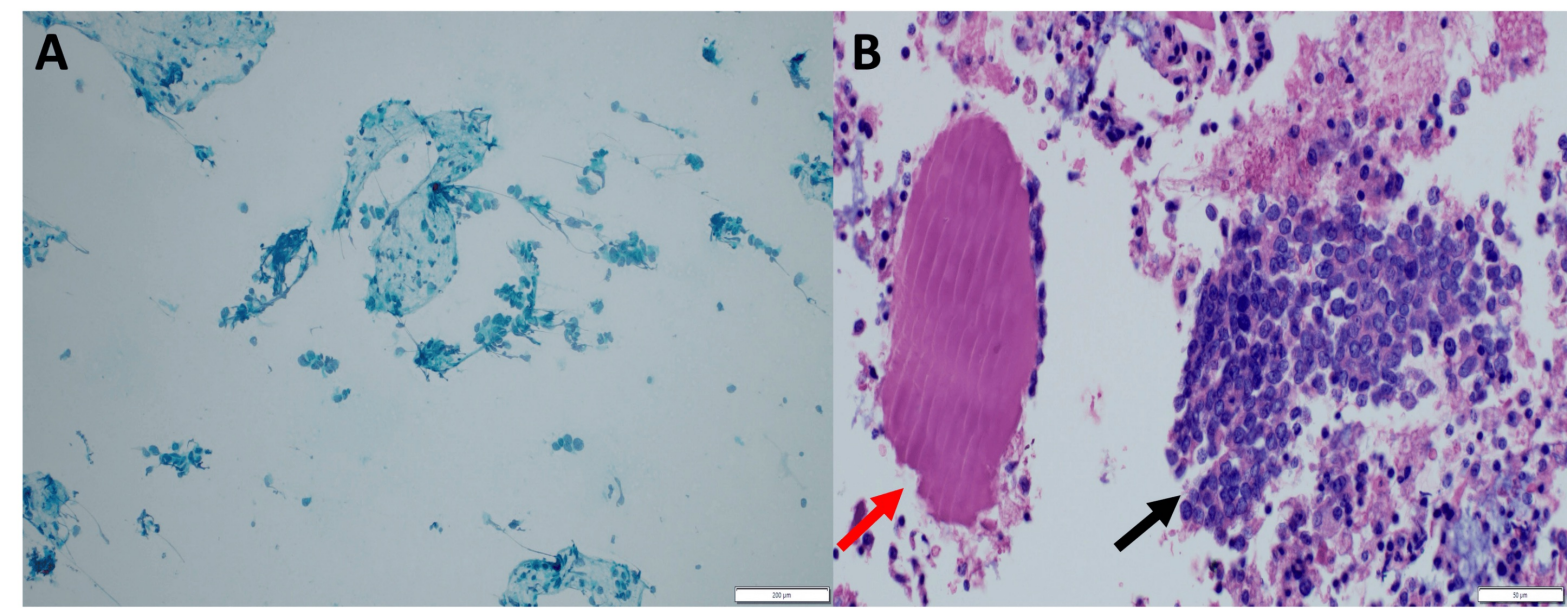
Cytology and cell block findings from thyroid metastasis. (A) Pap-stained cytology smear of the thyroid (10× objective) shows scattered aggregates and individual malignant cells. (B) Corresponding cell block section (40× objective) demonstrates the same malignant cells (black arrow). Adjacent colloid is visible (red arrow).

**Figure 6:**
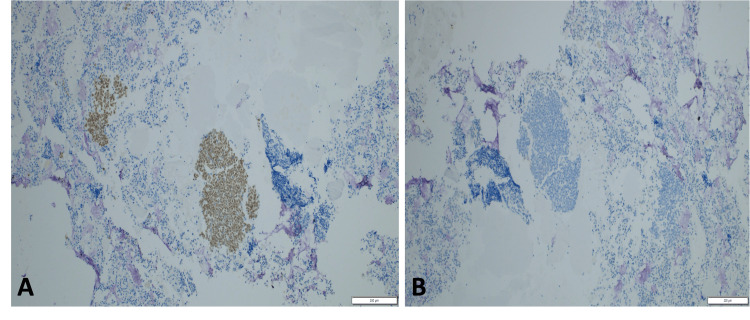
Immunohistochemical profile of metastatic breast carcinoma in the thyroid. (A) Tumor cells showing diffuse nuclear positivity for GATA-3 (10× objective), supporting a breast origin. (B) Tumor cells are negative for the thyroid marker PAX-8 (10× objective), confirming the lesion is metastatic rather than primary thyroid carcinoma. GATA-3: GATA binding protein 3 transcription factor; PAX-8: Paired Box 8 transcription factor

Imaging revealed multiple suspicious left breast lesions classified as BI-RADS 5, suggestive of multifocal, multicentric malignancy. Core biopsy confirmed invasive ductal carcinoma. Whole-body ^18^F-FDG PET/CT demonstrated uptake in the left breast mass and diffuse uptake in an enlarged, heterogeneous thyroid gland (Figure 4). Thyroid ultrasound revealed heterogeneous echotexture, a 9 mm solid nodule in the left thyroid lobe (Figure 4), and multiple enlarged left cervical and supraclavicular lymph nodes. Fine needle aspiration cytology of the thyroid nodule and lymph nodes showed malignant cells with cytomorphology and immunohistochemistry consistent with metastasis from breast carcinoma (Figures 5, 6).

Case 3: gastric metastasis

A 68-year-old woman presented with a history of treated right breast carcinoma on ongoing adjuvant hormonal therapy. She reported early satiety and nausea which was debilitating.

**Figure 7:**
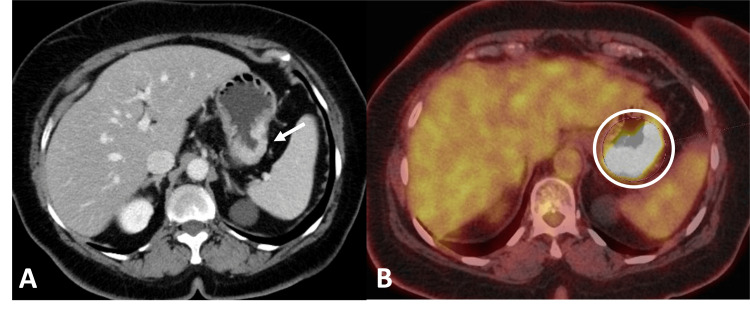
Breast cancer metastasis to the stomach. (A) Axial contrast-enhanced CT showing infiltrative mural thickening and hyperenhancement involving the greater curvature of the stomach (arrow). (B) Axial 18F-FDG PET-CT demonstrating intense FDG uptake (circle) corresponding to the area of gastric wall thickening and hyperenhancement. ^18^F-FDG PET-CT: Fluorine-18 fluorodeoxyglucose positron emission tomography/computed tomography; CT: Computed tomography; FDG: Fluorodeoxyglucose

**Figure 8:**
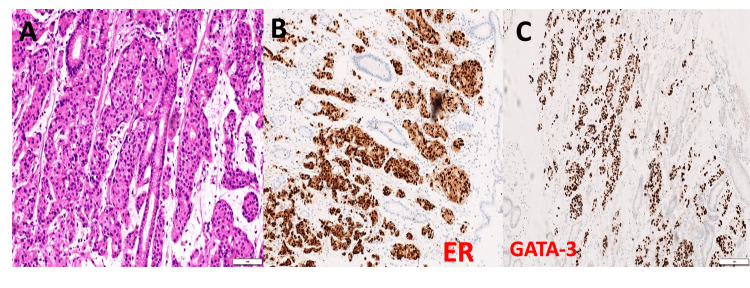
Metastatic invasive ductal carcinoma of the breast, NST, involving the stomach. (A) Hematoxylin and eosin (H&E) stain, 20× magnification, showing expansion of the lamina propria by nests of neoplastic epithelial cells. (B) Estrogen receptor (ER) immunohistochemistry demonstrating diffuse, strong nuclear staining of the neoplastic cells. (C) GATA-3 immunohistochemistry with diffuse nuclear positivity in the tumor cells. NST: No special type; GATA-3: GATA binding protein 3 transcription factor

^18^F-FDG PET/CT demonstrated intense curvilinear uptake in a thickened gastric wall along the greater curvature (Figure 7). Esophagogastroduodenoscopy revealed an ulcerating proliferative lesion. Endoscopic biopsy showed adenocarcinoma with immunohistochemical features favoring metastatic breast carcinoma (Figure 8). GATA-3 staining was strongly and diffusely positive in tumor cells (Figure 8), confirming breast origin.

Case 4: colonic metastasis

A 50-year-old woman was diagnosed with right breast invasive ductal carcinoma, staged T4N3M1. She underwent a right modified radical mastectomy, axillary lymph node dissection, and latissimus dorsi flap reconstruction, followed by adjuvant chemoradiotherapy (CHOP regimen). The patient was subsequently maintained on regular surveillance with PET-CT scans.

**Figure 9:**
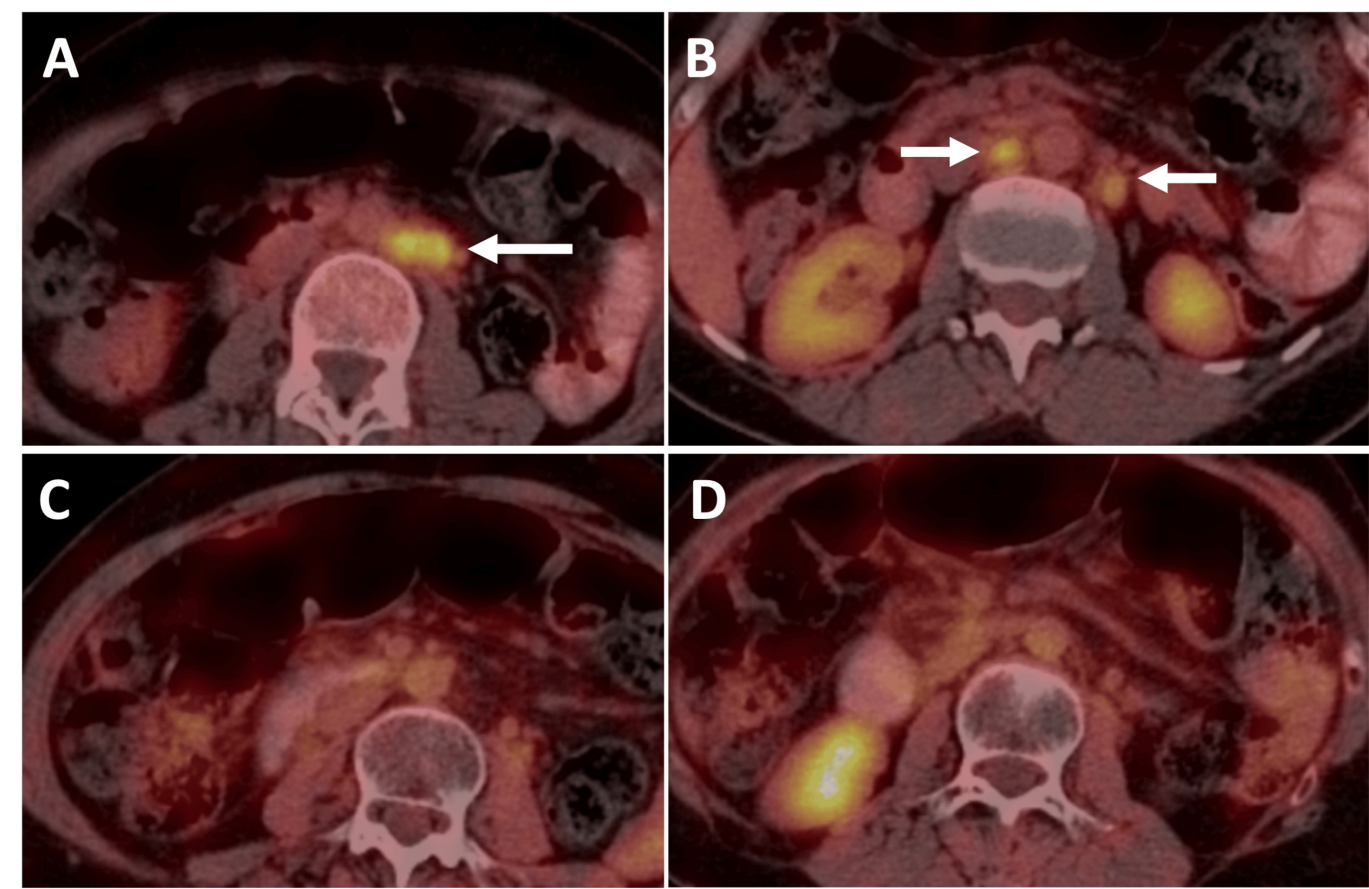
Metabolically active retroperitoneal lymphadenopathy in a patient with breast cancer. (A) Axial 18F-FDG PET-CT demonstrating FDG-avid left para-aortic lymph nodes (arrow). (B) Higher axial images showing additional retroperitoneal lymph nodes with increased FDG uptake (arrows). (C, D) Follow-up PET-CT after systemic chemoradiotherapy showing complete resolution of the previously identified FDG-avid retroperitoneal lymph nodes. ^18^F-FDG PET-CT: Fluorine-18 fluorodeoxyglucose positron emission tomography/computed tomography; CT: Computed tomography; FDG: Fluorodeoxyglucose

**Figure 10:**
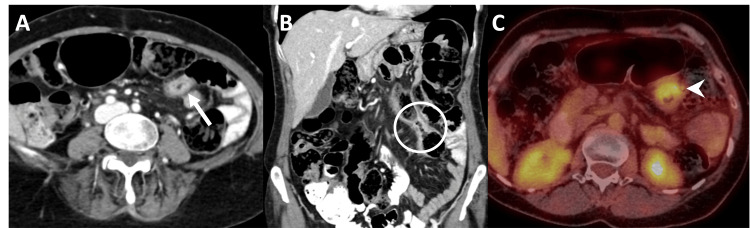
Breast cancer metastasis to the colon. (A) Axial contrast-enhanced CT showing infiltrative mural thickening and hyperenhancement of the transverse colon (arrow). (B) Coronal contrast-enhanced CT demonstrating the same lesion (circle). (C) Axial 18F-FDG PET-CT showing increased FDG uptake (arrowhead) corresponding to the colonic wall thickening and hyperenhancement. ^18^F-FDG PET-CT: Fluorine-18 fluorodeoxyglucose positron emission tomography/computed tomography; CT: Computed tomography; FDG: Fluorodeoxyglucose

**Figure 11:**
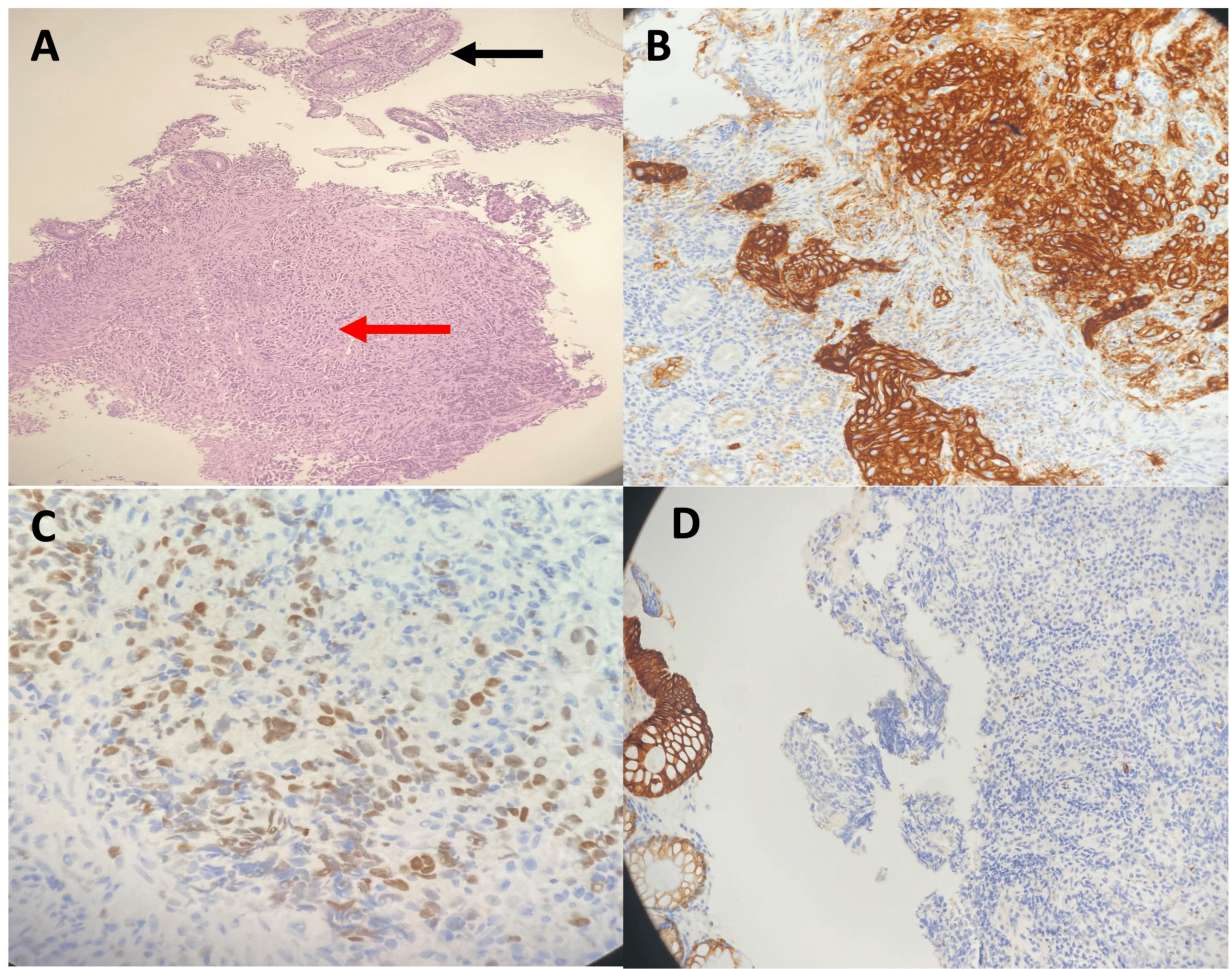
Histopathology and immunoprofile of metastatic breast carcinoma in the colon. (A) Hematoxylin and eosin-stained slide (10×) showing colonic mucosa (black arrow) with extensive submucosal infiltration by poorly differentiated adenocarcinoma (red arrow). (B) Tumor cells are positive for Cytokeratin-7 immunostain (20×). (C) Tumor cells exhibit multifocal weak to moderate nuclear positivity for GATA-3 immunostain (20×). (D) Tumor cells are negative for Cytokeratin-20 (10×), which highlights the normal colonic mucosa at the edge of the field. GATA-3: GATA binding protein 3 transcription factor

During follow-up, PET-CT revealed enlarged, metabolically active retroperitoneal lymph nodes concerning for metastasis (Figure 9). The patient continued on systemic chemotherapy and radiotherapy. Subsequent PET-CT demonstrated interval resolution of the retroperitoneal lymphadenopathy (Figure 9) but revealed new FDG-avid focal/segmental mural thickening in the left transverse colon (Figure 10), raising concern for either metastatic disease or a primary colorectal carcinoma. Colonoscopy showed a focal short segment stricture with circumferential polypoidal growth and mucosal irregularity in the left transverse colon. Biopsy was performed, and histopathological examination revealed poorly differentiated carcinoma. Immunohistochemistry demonstrated tumor cells positive for CK7 and GATA-3, consistent with metastatic breast carcinoma (Figure 11). The patient underwent laparoscopic left hemicolectomy. Histopathological examination of the resected specimen confirmed metastatic breast carcinoma. Tumor cells were positive for HER2, indicating eligibility for HER2-targeted therapy (Figure 11).

## Discussion

Breast carcinoma most commonly metastasizes to the bones, lungs, liver, and brain. Metastatic involvement of atypical sites, such as the parotid gland, thyroid gland, stomach, and colon, is exceedingly rare and can present significant diagnostic challenges [1-3]. Recognition of these uncommon metastatic patterns is essential to avoid misdiagnosis and inappropriate therapeutic interventions.

Metastatic disease to the parotid gland typically originates from head and neck primaries, particularly squamous cell carcinoma and malignant melanoma, whereas distant metastases from breast, renal, gastrointestinal, or prostate malignancies are distinctly uncommon [4,5]. Approximately 57 cases of breast cancer metastasis to the parotid gland have been reported, with hematogenous spread considered the most likely mechanism [5-8]. Most patients present with a painless parotid mass, occurring either synchronously or metachronously with the breast primary [7]. Our case is notable not only for contralateral parotid involvement but also for the parotid mass being the initial presenting symptom, preceding breast cancer diagnosis. Such presentations can easily be mistaken for primary salivary gland neoplasms, highlighting the importance of considering metastasis in the differential diagnosis, particularly in patients with known or suspected breast malignancy.

Despite its abundant vascularity, the thyroid gland is an infrequent site of metastatic disease, possibly due to its unique physiological environment, including high iodine and oxygen content [9]. Reported rates of thyroid involvement among secondary thyroid tumors range from 3% to 34% [9]. Invasive ductal carcinoma is more commonly associated with thyroid metastasis than lobular or metaplastic subtypes [10]. Approximately 59 cases of thyroid metastasis from breast cancer have been documented, with many identified incidentally on imaging performed for staging or surveillance [9,10]. Imaging findings may include focal or diffuse glandular enlargement and hypermetabolic activity on ^18^F-FDG PET/CT; however, definitive diagnosis relies on histopathology and immunohistochemistry. Expression of GATA-3 supports a breast origin, while the absence of PAX-8 helps exclude primary thyroid carcinoma [11,12].

Gastrointestinal tract (GIT) metastasis from breast cancer is similarly rare, comprising less than 1% of metastatic cases [2]. Invasive lobular carcinoma shows a greater predilection for GIT involvement, although ductal carcinoma can also metastasize to the gastrointestinal system [2]. The stomach and colon are the most frequently affected sites, typically presenting with diffuse submucosal or mural thickening and characteristic imaging features such as linitis plastica [2]. Gastric metastases can closely mimic primary gastric adenocarcinoma on clinical, endoscopic, and radiologic evaluation, making histologic and immunohistochemical confirmation critical [13,14]. Approximately 28% of gastrointestinal metastases from breast cancer involve the stomach [14], yet diagnostic yield on gastric biopsy may be low due to overlapping morphologic features [15,16].

Colonic metastasis is even rarer. McLemore et al. [17] identified colonic involvement in only 24 of 12,001 patients (0.2%) with metastatic breast cancer, while Taal et al. [18] reported 17 cases over 15 years. Colonic metastases, particularly from invasive ductal carcinoma, may closely resemble primary colorectal cancer, further complicating diagnosis [16]. Immunohistochemistry remains essential, guiding both diagnostic clarification and systemic therapy selection, including HER2-directed treatments when indicated [19,20]. Surgical intervention is generally reserved for palliation or management of complications such as obstruction or bleeding.

Although ^18^F-FDG PET/CT is valuable for identifying hypermetabolic lesions, it lacks specificity for differentiating primary from metastatic disease at uncommon sites. Several promising and novel radiotracers are being studied for PET/CT and imaging of Breast carcinoma and metastases. One such radiotracer is 18F-fluoroestradiol (18F-FES) which selectively binds to ER-α, making it well suited for imaging the ~80% of breast cancers that are ER-positive. Compared with ^18^F-FDG, FES shows minimal background uptake and offers greater specificity for ER+ disease. In a study at the Fudan University Shanghai Cancer Center [21], 19 patients underwent both scans across 245 total lesions; 41 lesions (16.7%) were detected only by 18F-FES and not by ^18^F-FDG. Moreover, FES PET shows higher sensitivity for detecting metastatic lesions than ^18^F-FDG PET, with reported rates of 90.8% versus 82.8%, respectively [21]. Another notable radiotracer is the fibroblast activation protein inhibitor (FAPI), which targets fibroblast activation protein (FAP), a type II transmembrane serine protease [22]. Comparative studies show that ⁶⁸Ga-FAPI demonstrates greater overall tumor uptake in breast cancer and detects metastatic disease more effectively than ¹⁸F-FDG, particularly for bone and peritoneal metastases [22]. A variety of radiotracers have been developed for imaging HER2-positive cancers, but ⁸⁹Zr-trastuzumab currently has the most robust supporting evidence [22]. Further research is necessary to rigorously evaluate the diagnostic performance, sensitivity, and specificity of these emerging tracers in comparison with conventional ¹⁸F-FDG PET/CT. Histopathology with immunohistochemistry remains the diagnostic gold standard. Expression of CK7 and GATA-3 strongly favors breast origin, while HER2 status provides critical information for therapeutic planning [22].

Management of metastases to rare sites is guided by disease burden and symptomatology. Systemic therapy remains the cornerstone of treatment and is tailored to the molecular characteristics of the primary tumor. Surgical intervention is typically reserved for symptom relief or local control. Early recognition of atypical metastatic patterns is crucial to avoid delays in diagnosis and unnecessary invasive procedures.

## Conclusions

This case series highlights the importance of considering metastatic breast carcinoma in the differential diagnosis of lesions at uncommon sites, even years after initial diagnosis or treatment. Metastatic involvement of the parotid gland, thyroid gland, and GIT, though rare, can closely mimic primary malignancies and often presents with nonspecific clinical or radiologic findings. Accurate diagnosis requires a comprehensive approach that integrates imaging, endoscopic evaluation, histopathology, and immunohistochemistry to confirm breast origin. Increased clinical awareness of these rare metastatic presentations can support timely diagnosis, guide appropriate systemic therapy, and help avoid unnecessary surgical interventions.
